# 4-Meth­oxy­benzaldehyde (5-bromo­pyrimidin-2-yl)hydrazone monohydrate

**DOI:** 10.1107/S1600536810033283

**Published:** 2010-08-28

**Authors:** Hoong-Kun Fun, Wan-Sin Loh, Suresh P. Nayak

**Affiliations:** a X-ray Crystallography Unit, School of Physics, Universiti Sains Malaysia, 11800 USM, Penang, Malaysia; b Department of Studies in Chemistry, Mangalore University, Mangalagangotri, Mangalore 574 199, India

## Abstract

In the title Schiff base compound, C_12_H_11_BrN_4_O·H_2_O, the organic mol­ecule exists in an *E* configuration with respect to the C=N double bond. The pyrimidine ring is approximately planar, with a maximum deviation of 0.011 (2) Å, and forms a dihedral angle of 10.68 (8)° with the benzene ring. In the crystal, inter­molecular O—H⋯N, N—H⋯O and C—H⋯O hydrogen bonds link the mol­ecules into a two-dimensional network parallel to the *ac* plane.

## Related literature

For the preparation of hydrazones, see: Pasha & Nanjundaswamy (2004 ▶). For the importance and biological activity of hydrazones, see: Sridhar & Perumal (2003 ▶); Rollas *et al.* (2002 ▶); Terzioglu & Gürsoy (2003 ▶). For the biological activity of pyrimidines and their derivatives, see: Ghorab *et al.* (2004 ▶). For a related structure, see: Zhang *et al.* (2009 ▶). For reference bond-length data, see: Allen *et al.* (1987 ▶). For the stability of the temperature controller used in the data collection, see: Cosier & Glazer (1986 ▶).
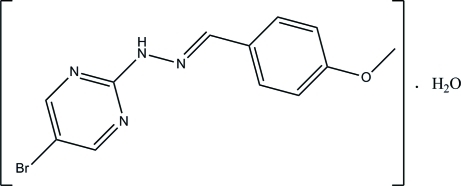


## Experimental

### 

#### Crystal data

C_12_H_11_BrN_4_O·H_2_O
*M* *_r_* = 325.17
Orthorhombic, 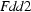

*a* = 13.0606 (3) Å
*b* = 60.5887 (10) Å
*c* = 6.5618 (1) Å
*V* = 5192.52 (17) Å^3^
*Z* = 16
Mo *K*α radiation
μ = 3.17 mm^−1^
*T* = 100 K
0.40 × 0.34 × 0.21 mm

#### Data collection

Bruker SMART APEXII CCD area-detector diffractometer
Absorption correction: multi-scan (*SADABS*; Bruker, 2009 ▶) *T*_min_ = 0.365, *T*_max_ = 0.558
12616 measured reflections
4593 independent reflections
4107 reflections with *I* > 2σ(*I*)
*R*_int_ = 0.029

#### Refinement

*R*[*F* ^2^ > 2σ(*F* ^2^)] = 0.026
*wR*(*F* ^2^) = 0.052
*S* = 0.92
4593 reflections
225 parameters
1 restraint
All H-atom parameters refined
Δρ_max_ = 0.43 e Å^−3^
Δρ_min_ = −0.40 e Å^−3^
Absolute structure: Flack (1983 ▶), 2037 Friedel pairs
Flack parameter: 0.012 (5)

### 

Data collection: *APEX2* (Bruker, 2009 ▶); cell refinement: *SAINT* (Bruker, 2009 ▶); data reduction: *SAINT*; program(s) used to solve structure: *SHELXTL* (Sheldrick, 2008 ▶); program(s) used to refine structure: *SHELXTL*; molecular graphics: *SHELXTL*; software used to prepare material for publication: *SHELXTL* and *PLATON* (Spek, 2009 ▶).

## Supplementary Material

Crystal structure: contains datablocks global, I. DOI: 10.1107/S1600536810033283/wn2404sup1.cif

Structure factors: contains datablocks I. DOI: 10.1107/S1600536810033283/wn2404Isup2.hkl

Additional supplementary materials:  crystallographic information; 3D view; checkCIF report

## Figures and Tables

**Table 1:** Hydrogen-bond geometry (Å, °)

*D*—H⋯*A*	*D*—H	H⋯*A*	*D*⋯*A*	*D*—H⋯*A*
O1*W*—H1*W*1⋯N2	0.84 (3)	2.55 (3)	3.153 (2)	131 (2)
O1*W*—H1*W*1⋯N4	0.84 (3)	2.30 (3)	3.0511 (19)	151 (2)
O1*W*—H2*W*1⋯N2^i^	0.84 (3)	2.01 (3)	2.8341 (19)	169 (2)
N3—H1*N*3⋯O1*W*^ii^	0.81 (3)	1.99 (3)	2.7773 (19)	165.1 (19)
C5—H5*A*⋯O1*W*^ii^	0.99 (2)	2.43 (2)	3.257 (2)	140.7 (13)

Symmetry codes: (i) 
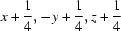
; (ii) 
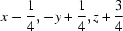
.

**Fig. 1.**
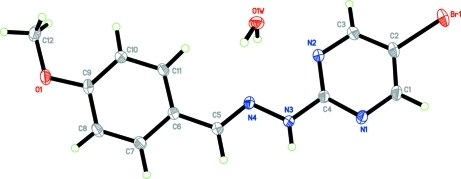
The molecular structure of the title compound, showing 50% probability displacement ellipsoids and the atom-numbering scheme. Hydrogen atoms are shown as spheres of arbitrary radius.

**Fig. 2.**
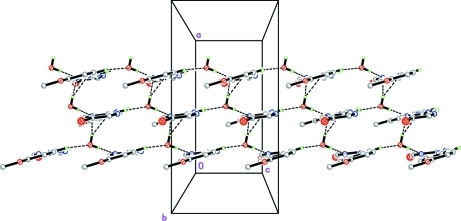
The crystal packing of the title compound, viewed along the b axis, showing the two-dimensional network parallel to the ac plane. H atoms not involved in the intermolecular interactions (dashed lines) have been omitted for clarity.

## supplementary crystallographic information

### Comment

Hydrazones have been prepared by treating aryl hydrazines with carbonyl compounds using a variety of solvents in the presence or absence of an acidic catalyst (Pasha & Nanjundaswamy, 2004). Aryl hydrazones are important building blocks for the synthesis of a variety of heterocyclic compounds such as pyrazolines and pyrazoles (Sridhar & Perumal, 2003). Hydrazones have been demonstrated to possess a variety of pharmacological activities (Rollas *et al.*, 2002; Terzioglu & Gürsoy, 2003). These observations have provided the guidelines for the development of new hydrazones that possess a variety of biological activities. Pyrimidines and their derivatives possess biological and pharmacological activities such as antibacterial, antimicrobial, anti-inflammatory, analgesic, anticonvulsant and anti-aggressive properties (Ghorab *et al.*, 2004). This prompted us to synthesize compounds containing the pyrimidine unit.

The asymmetric unit of the title Schiff base compound (Fig. 1) consists of one molecule of *p*-anisyl-(5-bromopyrimidin-2-yl)hydrazone and one water molecule. The *p*-anisyl-(5-bromopyrimidin-2-yl)hydrazone molecule exists in an *E* configuration with respect to the C5═N4 double bond. The pyrimidine ring (C1–C3/N2/C4/N1) is approximately planar, with a maximum deviation of 0.011 (2) Å at atom C3 and it forms a dihedral angle of 10.68 (8)° with the benzene ring (C6–C11). Bond lengths (Allen *et al.*, 1987) and angles are within the normal ranges and are comparable to those in the related crystal structure (Zhang *et al.*, 2009).

In the crystal packing (Fig. 2) intermolecular O1W—H1W1···N2, O1W—H1W1···N4 O1W—H2W1···N2, N3—H1N3···O1W and C5—H5A···O1W hydrogen bonds (Table 1) link the molecules into two-dimensional networks parallel to the *ac* plane.

### Experimental

The title compound was obtained by refluxing 5-bromo-2-hydrazinopyrimidine (0.01 mol) and 4-methoxybenzaldehyde (0.01 mol) in ethanol (30 ml), with the addition of 3 drops of concentrated sulfuric acid over a period of 1 h. Excess ethanol was removed from the reaction mixture under reduced pressure. The resulting solid product was filtered, washed with ethanol and dried. Colourless single crystals suitable for X-ray analysis were obtained from the ethanol solution by slow evaporation.

### Refinement

All H atoms were located in a difference Fourier map and were refined freely [C—H = 0.89 (2) to 1.03 (2) Å; N—H = 0.81 (3) Å; O—H = 0.83 (3) and 0.84 (3) Å].

### Crystal data

**Table:**

C_12_H_11_BrN_4_O·H_2_O	*F*(000) = 2624
*M**_r_* = 325.17	*D*_x_ = 1.664 Mg m^−^^3^
Orthorhombic, *F**d**d*2	Mo *K*α radiation, λ = 0.71073 Å
Hall symbol: F 2 -2d	Cell parameters from 5163 reflections
*a* = 13.0606 (3) Å	θ = 3.7–32.2°
*b* = 60.5887 (10) Å	µ = 3.17 mm^−^^1^
*c* = 6.5618 (1) Å	*T* = 100 K
*V* = 5192.52 (17) Å^3^	Block, colourless
*Z* = 16	0.40 × 0.34 × 0.21 mm

### Data collection

**Table:**

Bruker SMART APEXII CCD area-detector diffractometer	4593 independent reflections
Radiation source: fine-focus sealed tube	4107 reflections with *I* > 2σ(*I*)
graphite	*R*_int_ = 0.029
φ and ω scans	θ_max_ = 32.8°, θ_min_ = 2.7°
Absorption correction: multi-scan (*SADABS*; Bruker, 2009)	*h* = −18→19
*T*_min_ = 0.365, *T*_max_ = 0.558	*k* = −92→91
12616 measured reflections	*l* = −9→9

### Refinement

**Table:**

Refinement on *F*^2^	Secondary atom site location: difference Fourier map
Least-squares matrix: full	Hydrogen site location: inferred from neighbouring sites
*R*[*F*^2^ > 2σ(*F*^2^)] = 0.026	All H-atom parameters refined
*wR*(*F*^2^) = 0.052	*w* = 1/[σ^2^(*F*_o_^2^) + (0.*P*)^2^] where *P* = (*F*_o_^2^ + 2*F*_c_^2^)/3
*S* = 0.92	(Δ/σ)_max_ < 0.001
4593 reflections	Δρ_max_ = 0.43 e Å^−^^3^
225 parameters	Δρ_min_ = −0.40 e Å^−^^3^
1 restraint	Absolute structure: Flack (1983), 2037 Friedel pairs
Primary atom site location: structure-invariant direct methods	Flack parameter: 0.012 (5)

### Special details

**Table:**

Experimental. The crystal was placed in the cold stream of an Oxford Cryosystems Cobra open-flow nitrogen cryostat (Cosier & Glazer, 1986) operating at 100.0 (1) K.
Geometry. All e.s.d.'s (except the e.s.d. in the dihedral angle between two l.s. planes) are estimated using the full covariance matrix. The cell e.s.d.'s are taken into account individually in the estimation of e.s.d.'s in distances, angles and torsion angles; correlations between e.s.d.'s in cell parameters are only used when they are defined by crystal symmetry. An approximate (isotropic) treatment of cell e.s.d.'s is used for estimating e.s.d.'s involving l.s. planes.
Refinement. Refinement of *F*^2^ against ALL reflections. The weighted *R*-factor *wR* and goodness of fit *S* are based on *F*^2^, conventional *R*-factors *R* are based on *F*, with *F* set to zero for negative *F*^2^. The threshold expression of *F*^2^ > σ(*F*^2^) is used only for calculating *R*-factors(gt) *etc*. and is not relevant to the choice of reflections for refinement. *R*-factors based on *F*^2^ are statistically about twice as large as those based on *F*, and *R*- factors based on ALL data will be even larger.

### Fractional atomic coordinates and isotropic or equivalent isotropic displacement parameters (Å^2^)

**Table:**

	*x*	*y*	*z*	*U*_iso_*/*U*_eq_	
Br1	0.088547 (13)	0.026408 (2)	0.69853 (3)	0.02305 (5)	
O1	0.08828 (10)	0.235769 (16)	0.7073 (2)	0.0206 (2)	
N1	0.04426 (12)	0.07710 (2)	1.1084 (2)	0.0181 (3)	
N2	0.05839 (12)	0.09353 (2)	0.7759 (2)	0.0168 (3)	
N3	0.03392 (13)	0.11442 (2)	1.0691 (2)	0.0175 (3)	
N4	0.04759 (10)	0.133332 (18)	0.9574 (2)	0.0156 (2)	
C1	0.05708 (15)	0.05747 (3)	1.0215 (3)	0.0194 (3)	
C2	0.07009 (14)	0.05472 (3)	0.8133 (3)	0.0182 (3)	
C3	0.06893 (12)	0.07344 (2)	0.6943 (3)	0.0176 (3)	
C4	0.04600 (12)	0.09446 (2)	0.9789 (2)	0.0149 (3)	
C5	0.03372 (14)	0.15154 (3)	1.0534 (3)	0.0165 (3)	
C6	0.04819 (11)	0.17289 (2)	0.9545 (3)	0.0147 (3)	
C7	0.03424 (14)	0.19218 (3)	1.0688 (2)	0.0183 (3)	
C8	0.04797 (14)	0.21285 (2)	0.9828 (2)	0.0186 (3)	
C9	0.07649 (14)	0.21461 (3)	0.7790 (2)	0.0167 (3)	
C10	0.09188 (14)	0.19569 (3)	0.6627 (2)	0.0167 (3)	
C11	0.07730 (14)	0.17500 (3)	0.7492 (2)	0.0167 (3)	
C12	0.11941 (17)	0.23826 (3)	0.5009 (3)	0.0242 (4)	
O1W	0.24687 (11)	0.12558 (2)	0.72556 (19)	0.0203 (3)	
H1W1	0.184 (2)	0.1255 (4)	0.749 (4)	0.037 (7)*	
H2W1	0.2655 (18)	0.1332 (4)	0.826 (4)	0.027 (6)*	
H1N3	0.0212 (17)	0.1149 (3)	1.189 (5)	0.030 (6)*	
H1A	0.0590 (17)	0.0449 (4)	1.110 (3)	0.023 (5)*	
H3A	0.0769 (19)	0.0728 (4)	0.546 (4)	0.035 (7)*	
H5A	0.0120 (15)	0.1507 (3)	1.198 (4)	0.022 (5)*	
H7A	0.0149 (15)	0.1910 (3)	1.218 (4)	0.020 (5)*	
H8A	0.041 (2)	0.2255 (4)	1.052 (4)	0.036 (7)*	
H10A	0.1133 (19)	0.1960 (4)	0.522 (4)	0.036 (6)*	
H11A	0.0919 (16)	0.1621 (3)	0.667 (4)	0.023 (5)*	
H12A	0.1909 (19)	0.2312 (3)	0.491 (4)	0.033 (6)*	
H12B	0.1262 (15)	0.2538 (3)	0.476 (4)	0.023 (5)*	
H12C	0.0727 (17)	0.2324 (4)	0.415 (4)	0.031 (6)*	

### Atomic displacement parameters (Å^2^)

**Table:**

	*U*^11^	*U*^22^	*U*^33^	*U*^12^	*U*^13^	*U*^23^
Br1	0.02252 (8)	0.01183 (6)	0.03480 (9)	0.00168 (6)	−0.00211 (8)	−0.00497 (7)
O1	0.0280 (6)	0.0115 (4)	0.0223 (5)	−0.0010 (5)	−0.0027 (5)	0.0006 (6)
N1	0.0205 (8)	0.0142 (6)	0.0195 (7)	0.0001 (5)	0.0023 (6)	0.0035 (5)
N2	0.0206 (7)	0.0128 (6)	0.0171 (6)	0.0004 (5)	0.0013 (5)	−0.0002 (5)
N3	0.0270 (8)	0.0122 (6)	0.0133 (6)	0.0003 (6)	0.0036 (6)	0.0007 (5)
N4	0.0195 (6)	0.0118 (5)	0.0156 (6)	−0.0014 (5)	0.0001 (6)	0.0011 (5)
C1	0.0193 (9)	0.0127 (7)	0.0261 (8)	−0.0008 (6)	0.0026 (7)	0.0035 (6)
C2	0.0152 (8)	0.0123 (7)	0.0273 (9)	−0.0005 (6)	0.0001 (6)	−0.0020 (6)
C3	0.0165 (8)	0.0152 (6)	0.0211 (7)	0.0015 (5)	0.0001 (8)	−0.0020 (7)
C4	0.0149 (7)	0.0123 (6)	0.0176 (8)	−0.0002 (5)	0.0011 (6)	0.0006 (5)
C5	0.0194 (9)	0.0138 (7)	0.0164 (7)	0.0013 (6)	0.0009 (6)	−0.0002 (5)
C6	0.0147 (7)	0.0125 (6)	0.0168 (6)	0.0011 (5)	−0.0007 (7)	−0.0007 (6)
C7	0.0226 (9)	0.0159 (7)	0.0164 (7)	0.0017 (6)	−0.0009 (6)	−0.0017 (6)
C8	0.0236 (8)	0.0127 (7)	0.0195 (8)	0.0020 (6)	−0.0029 (7)	−0.0040 (6)
C9	0.0180 (8)	0.0106 (7)	0.0216 (7)	−0.0002 (6)	−0.0041 (6)	0.0006 (5)
C10	0.0195 (8)	0.0152 (6)	0.0154 (8)	−0.0006 (6)	0.0004 (6)	−0.0011 (5)
C11	0.0210 (9)	0.0127 (6)	0.0164 (8)	0.0019 (6)	−0.0006 (6)	−0.0021 (5)
C12	0.0258 (10)	0.0161 (7)	0.0307 (10)	−0.0008 (7)	0.0068 (8)	0.0045 (6)
O1W	0.0256 (7)	0.0202 (5)	0.0152 (6)	−0.0051 (5)	0.0025 (6)	−0.0026 (5)

### Geometric parameters (Å, °)

**Table:**

Br1—C2	1.8886 (17)	C5—H5A	0.99 (2)
O1—C9	1.3745 (19)	C6—C7	1.401 (2)
O1—C12	1.422 (2)	C6—C11	1.406 (2)
N1—C1	1.329 (2)	C7—C8	1.386 (2)
N1—C4	1.352 (2)	C7—H7A	1.01 (2)
N2—C3	1.337 (2)	C8—C9	1.392 (2)
N2—C4	1.343 (2)	C8—H8A	0.89 (2)
N3—C4	1.356 (2)	C9—C10	1.392 (2)
N3—N4	1.3719 (18)	C10—C11	1.389 (2)
N3—H1N3	0.81 (3)	C10—H10A	0.96 (2)
N4—C5	1.284 (2)	C11—H11A	0.97 (2)
C1—C2	1.387 (3)	C12—H12A	1.03 (2)
C1—H1A	0.95 (2)	C12—H12B	0.957 (19)
C2—C3	1.377 (2)	C12—H12C	0.90 (3)
C3—H3A	0.98 (3)	O1W—H1W1	0.83 (3)
C5—C6	1.459 (2)	O1W—H2W1	0.84 (3)
			
C9—O1—C12	117.21 (13)	C11—C6—C5	122.81 (14)
C1—N1—C4	115.10 (14)	C8—C7—C6	121.30 (15)
C3—N2—C4	116.63 (14)	C8—C7—H7A	119.3 (9)
C4—N3—N4	119.77 (14)	C6—C7—H7A	119.4 (9)
C4—N3—H1N3	118.9 (14)	C7—C8—C9	119.66 (14)
N4—N3—H1N3	121.3 (14)	C7—C8—H8A	123.6 (17)
C5—N4—N3	115.91 (15)	C9—C8—H8A	116.8 (17)
N1—C1—C2	123.06 (16)	O1—C9—C10	124.35 (15)
N1—C1—H1A	117.1 (13)	O1—C9—C8	115.47 (14)
C2—C1—H1A	119.8 (13)	C10—C9—C8	120.17 (14)
C3—C2—C1	117.26 (16)	C11—C10—C9	119.95 (14)
C3—C2—Br1	121.57 (14)	C11—C10—H10A	116.7 (14)
C1—C2—Br1	121.17 (14)	C9—C10—H10A	123.3 (14)
N2—C3—C2	121.59 (19)	C10—C11—C6	120.72 (14)
N2—C3—H3A	116.5 (15)	C10—C11—H11A	118.3 (13)
C2—C3—H3A	121.9 (15)	C6—C11—H11A	120.9 (13)
N2—C4—N1	126.34 (14)	O1—C12—H12A	105.9 (13)
N2—C4—N3	118.98 (13)	O1—C12—H12B	106.9 (14)
N1—C4—N3	114.68 (14)	H12A—C12—H12B	108.1 (17)
N4—C5—C6	121.68 (15)	O1—C12—H12C	111.1 (15)
N4—C5—H5A	117.8 (10)	H12A—C12—H12C	114 (2)
C6—C5—H5A	120.5 (10)	H12B—C12—H12C	110 (2)
C7—C6—C11	118.19 (14)	H1W1—O1W—H2W1	98 (2)
C7—C6—C5	118.99 (16)		
			
C4—N3—N4—C5	179.31 (15)	N4—C5—C6—C7	−178.50 (16)
C4—N1—C1—C2	−0.8 (3)	N4—C5—C6—C11	0.4 (3)
N1—C1—C2—C3	−0.3 (3)	C11—C6—C7—C8	0.4 (3)
N1—C1—C2—Br1	179.74 (14)	C5—C6—C7—C8	179.38 (16)
C4—N2—C3—C2	−1.9 (2)	C6—C7—C8—C9	−0.2 (3)
C1—C2—C3—N2	1.7 (3)	C12—O1—C9—C10	−0.7 (2)
Br1—C2—C3—N2	−178.33 (12)	C12—O1—C9—C8	178.75 (17)
C3—N2—C4—N1	0.8 (3)	C7—C8—C9—O1	180.00 (15)
C3—N2—C4—N3	−179.34 (15)	C7—C8—C9—C10	−0.5 (3)
C1—N1—C4—N2	0.6 (3)	O1—C9—C10—C11	−179.58 (15)
C1—N1—C4—N3	−179.35 (16)	C8—C9—C10—C11	0.9 (3)
N4—N3—C4—N2	−8.4 (2)	C9—C10—C11—C6	−0.7 (3)
N4—N3—C4—N1	171.56 (15)	C7—C6—C11—C10	0.1 (2)
N3—N4—C5—C6	178.45 (14)	C5—C6—C11—C10	−178.87 (16)

### Hydrogen-bond geometry (Å, °)

**Table:**

*D*—H···*A*	*D*—H	H···*A*	*D*···*A*	*D*—H···*A*
O1W—H1W1···N2	0.84 (3)	2.55 (3)	3.153 (2)	131 (2)
O1W—H1W1···N4	0.84 (3)	2.30 (3)	3.0511 (19)	151 (2)
O1W—H2W1···N2^i^	0.84 (3)	2.01 (3)	2.8341 (19)	169 (2)
N3—H1N3···O1W^ii^	0.81 (3)	1.99 (3)	2.7773 (19)	165.1 (19)
C5—H5A···O1W^ii^	0.99 (2)	2.43 (2)	3.257 (2)	140.7 (13)

Symmetry codes: (i) *x*+1/4, −*y*+1/4, *z*+1/4; (ii) *x*−1/4, −*y*+1/4, *z*+3/4.
